# An open database of productivity in Vietnam's social sciences and humanities for public use

**DOI:** 10.1038/sdata.2018.188

**Published:** 2018-09-25

**Authors:** Quan-Hoang Vuong, Viet-Phuong La, Thu-Trang Vuong, Manh-Toan Ho, Hong-Kong T. Nguyen, Viet-Ha Nguyen, Hiep-Hung Pham, Manh-Tung Ho

**Affiliations:** 1 Thanh Tay University, Centre for Interdisciplinary Social Research, Yen Nghia Ward, Ha Dong District, Hanoi 100000, Vietnam; 2 Université Libre de Bruxelles, Centre Emile Bernheim, Brussels, Belgium; 3 Phu Xuan University, Hue City, 530000, Vietnam; 4 Sciences Po Paris, Campus de Dijon, Dijon 21000, France; 5 Vietnam Panorama Media Monitoring, D5 Giang Vo, Hanoi 100000, Vietnam; 6 Vietnam Academy of Social Sciences, Institute of Philosophy, No. 59, Lang Ha Street, Thanh Cong Ward, Ba Dinh District, Hanoi 100000, Vietnam; 7 Ritsumeikan Asia Pacific University, Beppu City, Oita Prefecture 874-8577, Japan

**Keywords:** Interdisciplinary studies, Databases, Policy, Developing world, Publishing

## Abstract

This study presents a description of an open database on scientific output of Vietnamese researchers in social sciences and humanities, one that corrects for the shortcomings in current research publication databases such as data duplication, slow update, and a substantial cost of doing science. Here, using scientists’ self-reports, open online sources and cross-checking with Scopus database, we introduce a manual system and its semi-automated version of the database on the profiles of 657 Vietnamese researchers in social sciences and humanities who have published in Scopus-indexed journals from 2008 to 2018. The final system also records 973 foreign co-authors, 1,289 papers, and 789 affiliations. The data collection method, highly applicable for other sources, could be replicated in other developing countries while its content be used in cross-section, multivariate, and network data analyses. The open database is expected to help Vietnam revamp its research capacity and meet the public demand for greater transparency in science management.

## Background & Summary

Building a sustainable scholar community is crucial to the sustainable development of a country^1,2^. Yet, for developing countries like Vietnam, the sustainability focus is frequently reduced to climate change adaptation or related environment issues. The construction of this dataset was motivated by the desire to engage scholars both in Vietnam and overseas in a new dialogue. An open dataset on the productivity of Vietnamese researchers in social sciences and humanities (SS&H) as well as their foreign colleagues can be very useful as the government and the public increasingly call for transparency of research funds and promotions of professors in the higher education system.

Studies of scientific activities, science communication and policy, known as scientometrics^3^, have been done throughout the world since the 1960s^4^. By comparison, Vietnam got a rather late start in the field—only until 2011 was there a study on the relationship between scientific output and knowledge economy within the Association of Southeast Asian Nations (ASEAN). Nguyen & Pham found that in the 1991–2010 period, the ASEAN countries published over 165,000 articles in journals indexed in the Web of Science (WoS) database, of which Vietnam contributed only 6% and ranked fourth in the region^5^. Similar studies in recent years, based on either the Scopus or WoS database, highlighted an uptrend in the output of Vietnamese researchers, with an annual growth of between 17% and 20% in the 2001–2015 period^6,7^. Yet, data also noted the prevalence of international collaboration, from 75% to 77%, in these publications^8,9^.

A common method was used in the aforementioned studies—relying entirely on data from Scopus and WoS, using key words such as ‘Vietnam’ or ‘ASEAN countries,’ and focusing only on journal articles^5–7^. However, there are three issues with this approach and these databases, namely: (i) duplicate data, (ii) delay in database update, and (iii) cost of doing science. For example, one study found 12% of the records in the seven Scopus-indexed journals to be duplicate^8^. Worse still, not only does it often take months for this kind of database to update new articles, it also presents a substantial cost for continuous database subscription. While no pricing figures for Scopus and Web of Science subscription are available to the public, one study estimated this at $100,000–$120,000 a year for large organizations^9^. Given the fierce competition for research funding and the demand for transparency in science management, annual subscription to this kind of database poses a considerable cost for researchers, especially those in transition economies like Vietnam^10^.

Against this background is Vietnam’s determination to improve research capacity. As its science policy-makers are embroiled in heated debates about the low-quality research in SS&H^11–15^, this calls for the creation of a comprehensive system that generates accurate, Vietnam-specific information on the productivity and demographic characteristics of local SS&H researchers with international publications. This study presents two systems, manual and semi-automatic, to collect and verify such information for the 2008–2018 period. Both methods start with collecting scientific profiles provided by researchers and published on websites of public institutions, followed by cross validation with free online resources such as journals’ websites, Google Scholar, Scimagojr, Scopus’s open data, etc.

The initial datasets resulted from this manual system are the basis for five publications. Three articles applied cross-section data analysis to study the trends in productivity associated with collaboration, gender, age, regions, and first-authorship of Vietnamese social scientists. The first showed no significant difference in international publications between Vietnamese male and female researchers in SS&H and a strong correlation between the age of authors in leading role and scientific output^16^. The second found that Vietnamese social scientists heavily relied on collaboration as non-leading authors: on average, they collaborated 13 times during 2008–2018 and 90% co-wrote a publication with other authors^17^. The third revealed the contribution-adjusted productivity (‘cp’) could be boosted by a ratio of 1:1.06 and most researchers with high ‘cp’ fall into the age group of 40–50 years old^18^. Two other articles employed network statistical analysis to examine collaboration patterns among Vietnamese social scientists and found: (i) insufficient information dissemination in the co-authorship network, (ii) networks dependency on a few highly connected members^11^, and (iii) some signs of unsustainability^2^.

This paper will explain the overall procedure, the shortcomings of the manual data collection system, and the operation of the semi-automatic version. The ultimate purpose is to improve the data quality control and to generate more varieties of data to serve future research directions.

As the problems posed by the Scopus original database are common in other scientific publications databases such as WoS, MathSciNet, and Pubmed, among others, our manual and semi-automatic systems will be highly applicable elsewhere.

## Methods

The database is built in accordance with the FAIR (Findability, Accessibility, Interoperability, and Reusability) principle, which is first espoused by Wilkinson *et al*.^19^.

### Overview

**Figure 1:**
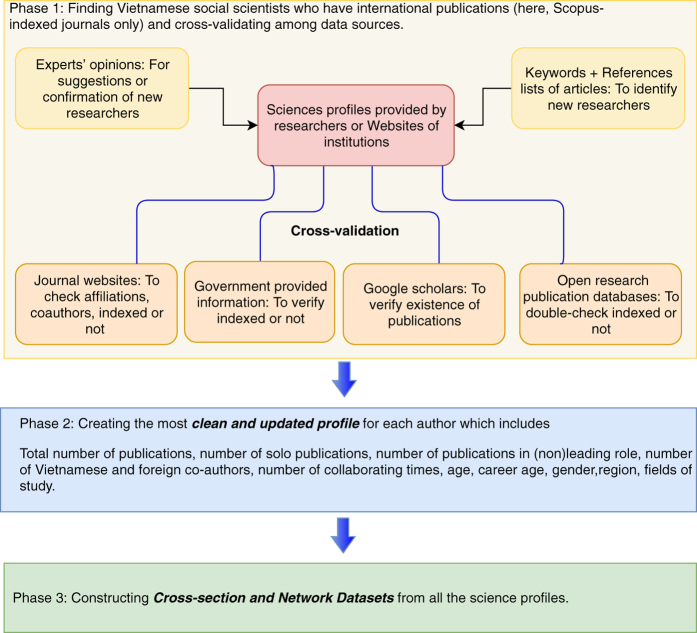
Project conceptualization. The project, from data collection to datasets construction, consists of three phases. Phase 1 is about identifying Vietnamese social scientists who have international publications and cross-checking among various data sources. Phase 2 is about creating a personal science profile for each author which includes information on the author’s scientific output and demographic characteristics. Phase 3 is about constructing cross-section and network datasets from all the science profiles.

The data collection process, summarized in Fig. 1, is comprised of three phases: searching, creating science profiles, and constructing master datasets. The goals are to:

- collect the data on every Vietnamese social sciences and humanities (SS&H) researcher who has published in Scopus-indexed journals from 2008 to 2018;
- ensure reliability and accuracy.

To achieve the goals, the search covers only Vietnamese researchers in SS&H of Vietnamese nationality that meet at least one of the following criteria:

- They are affiliated with an organization in Vietnam; OR
- They have published at least one paper about Vietnam or use data collected in Vietnam related to SS&H topics.

The search is further confined to Vietnamese authors who have published in Scopus-indexed scientific journals. It is important to note that the method could in principle cover publications indexed in the WoS, MathSciNet, PubMed and other reliable scientific databases. For comparison purpose, Scopus indexed about 22,600 titles^20^, which is almost twice as many as its counterpart WoS^21^. Given the project aims to serve Vietnamese science policymakers, we take into account the fact that Scopus is one of many scholarly databases used by the Vietnamese government to judge academic credentials^22^. Specifically, in a governmental decision, the Vietnam National Foundation for Science & Technology Development (henceforth referred to as NAFOSTED), Vietnam’s leading funder for science and technology research, has provided a list of prestigious international and national journals in the field of SS&H, which includes being indexed in Scopus as a criteria^23^. This is also a common practice in various countries including the United States, Spain, and Russia^24–26^ as well as for highly influential rankings such as the Times Highers Education^27,28^.

Based on these basic principles, next we will delve into the manual data collection system, its procedure and shortcomings that prompt the need for the semi-automatic system.

### NVSS Manual System

The manual process of data collection and verification was carried out from 1^st^ February 2017 to 15^th^ July 2017, which resulted in the creation of the Network of Vietnamese Social Scientists (NVSS) dataset. NVSS contains 412 science profiles for 412 distinct Vietnamese researchers in social sciences and humanities who have published in Scopus-indexed journals. An example of these first science profiles could be found in Data Citation 1.

### Procedure

The first step of the data collection process was to access websites of research institutions in Vietnam to identify researchers who fit the above criteria. Then, based on their public CVs, we marked down the number of publications they have authored and their demographic information. Next, we cross-checked these newly gathered data with websites of journals, Google Scholars, Scimagojr, and Scopus to make sure the information claimed on the CVs was in fact accurate. The Scopus system, therefore, has only value for us to double-check by examining if a randomly chosen research item has been present in their indexing system.

To ensure that the manual process covers as many eligible Vietnamese researcher as possible, we also looked at the references lists of the articles and experts’ opinions, as well as used varied keywords (‘Vietnamese economic development’, ‘Vietnamese history’, ‘Vietnamese culture’, etc.), and other resources such as social media, online news outlets, to name a few. The experts are from organizations such as the State Council for Professor title of Vietnam; the Scientific Committees of NAFOSTED; other scientific boards of leading research institutions such as national universities; Vietnam Academy of Social Sciences; etc. or others with long-term experiences or high productivity in their respective disciplines. In the data collection stage, our team members would reach out to the experts for suggestions or confirmation of eligible researchers, then subject these suggestions to the rigorous cross-validation process.

**Figure 2:**
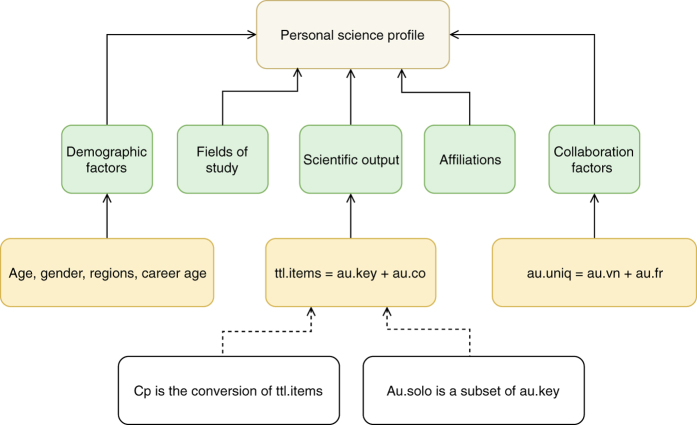
The relationship among variables recorded in this study. A personal science profile consists of five groups of factors: scientific output, demographic factors, collaboration factors, fields of study and affiliation. Scientific output factors concern with total number of publications, solo publications, publications in leading (key) position, and contribution-adjusted productivity. Demographic factors include age, gender, regions, and career age. Collaboration factors concern with total number of collaborators, of domestic collaborators, and of foreign collaborators. Two other factors are fields of study and affiliations.

The second step was to create a personal science profile for each Vietnamese author. Each said science profile corresponded to 13 lines of data (see Table 1). This process resulted in a clean, concise dataset of the most updated and complete profiles. We then contacted and invited the researchers to corroborate the profiles made by our team; the examples of some corroborated profiles could be found in Data Citation 1’s Scientific Profiles (Examples) folder. A list of input names and explanations appears in Table 1 while their relationships are illustrated in Fig. 2.

The third step involved summarizing all the profiles into a master file. The example of the master file resulted from the manual system could be found in Ho *et al*.^2,9^.

### Shortcomings

This manual method, albeit rigorous, faces two major shortcomings. First, the manual input of data is time-consuming and rigid, thus prone to human errors. The resulting dataset enables us to count how many publications each author has but lacks the capability for counting how many unique publications and journals exist in the entire database. This loophole excludes us from answering important questions such as how many new articles Vietnamese social scientists produce each year; or from generating data on international co-authorship network. Second, because the contribution-adjusted productivity (‘cp’) was computed manually, it would be immensely costly to switch to a different counting method such as the norm of all authors getting an equal share or the norm of first-last emphasis^29,30^.

### SSHPA Semi-automatic system

The semi-automatic system, called Social Sciences & Humanities Peer Awards (SSHPA), was kicked off on 1^st^ December 2017 and wrapped up on 2^nd^ February 2018 to resolve problems posed in the manual process. The purpose was to have a system capable of: (i) validating the quality of data previously collected, and (ii) making our database more flexible, less time-consuming to construct, and less prone to human errors. The semi-automated process also enables us to cover as close as possible to the actual number of eligible Vietnamese social scientists. For a brief overview of the distribution by sex, there were 262 female (39.88%) and 391 male researchers (59.51%), with four left unknown. Table 2 shows the descriptive statistics for continuous variables used in the SSHPA system. Other datasets related to these statistics can also be viewed in Data Citation 1’s Extracted and Computed Data’s table.

### System architecture

The SSHPA system, accessible online at https://sshpa.com/, is structured in MS SQL Server 2012 and is indexed to search Fulltext to centralize the management process. Its architecture is organized according to Client-server architecture. The software Server is built using Net Core which provides the APIs connections and functional modules such as Data Search & Filters, Data Validation, Network Builder and Reports. In addition, SSHPA Client software is built with C# that connects the database server through REST API Interface, this is intended to provide the users with complete data-input and data-check functions.

**Figure 3:**
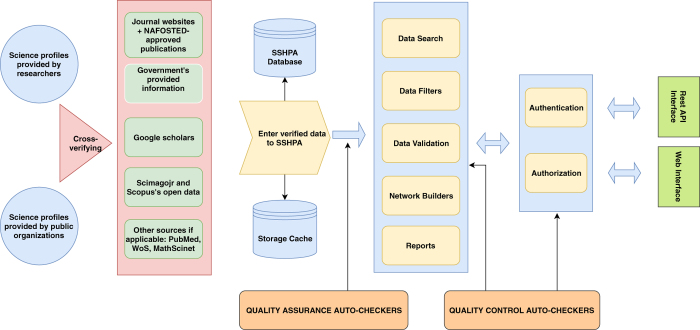
The system architecture of SSHPA. The system consists essentially of three major steps: (i) collecting the profiles of social scientists and cross-verifying with five other sources, (ii) entering the verified data into the SSHPA database and getting checked by the automated quality assurance, after data are in the system, the quality control auto-checkers would screen the database again for consistency and accuracy, and (iii) authenticating and authorizing (through three levels of admins, supervisors, collectors) the final science profiles in the SSHPA database.

Similar to the manual data collection process, the first step was to search for profiles of Vietnamese social scientists fitting our criteria. As shown in Fig. 3, we collected the profiles provided by researchers and organizations then verified with other sources such as government websites, NAFOSTED’s designated publications, journal websites, Scimagojr, Google Scholars, Scopus’ freely accessible data, etc.

The verified data were then entered into the SSHPA database and put through automated quality assurance and quality control steps. SSHPA was also designed with an authorization system with three levels: admins, supervisors, and collectors. Collectors could only input and edit unapproved data. Supervisors could approve a data entry, however, once the data entry is approved as most complete and accurate by the judgement of the supervisors, it cannot be changed or removed by either the supervisors or the collectors. Only the admins could remove a data entry or unlock the approved data for changes. Hence, in each level of authorization, each person must be accountable for the accuracy and reliability of the data entered into the system. With the nature of being semi-automated, SSHPA was still prone to human errors; this authorization mechanism was a way to uncover the mistakes in a timely manner, and thus, minimizing the consequences.

### Data structure

The data, once entered into our system, were organized in table structure in RDBMS.

**Figure 4:**
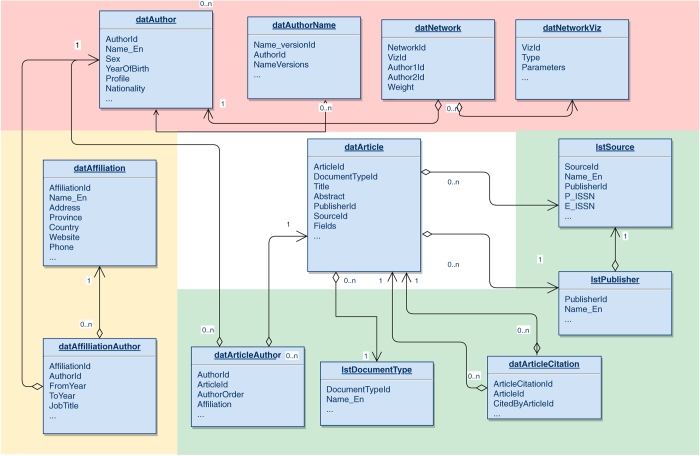
SSHPA’s data structure diagram: relationships among authors, articles, affiliations, fields, sources, and publishers. These are four kinds of data in SSHPA system and they are related to each other through one fundamental unit—*datArticle.* The pink block contains boxes pertaining to the authors and their networks information. The green block contains boxes pertaining to the sources, publishers and articles information. The yellow block contains boxes pertaining to the authors’ affiliations.

We designate Article as the fundamental unit of SSHPA’s data structure (center of Fig. 4), because: (i) an article’s name is often long enough to reduce the odds of data duplication, and (ii) an article published on a journal’s website will provide the other information such as authors, authors’ affiliation, publication year, and so on. This means all the other kinds of data: Author, Affiliation, Source, Publisher, Network, etc. are connected through Article.

For example, the *datArticle* box and the *datAuthor* box are connected to each other through an intermediary, *datArticleAuthor*, which holds information that connects the authors with their publications such as: author IDs, article ID, order of the author(s), affiliations of the authors, etc. The *datArticle* box contains the relevant data on the articles or publications in the database: title, document type (proceedings or journal articles for example), publisher ID, journal ID, etc. The data are fed from other boxes which contain information on the publishers (*lstPulisher*), the sources (journals or proceedings of conferences or books) of the articles (*lstSource*), the citation information (*lstCitation*), or the document type (*lstDocumentType*). Similar principles are applied to Network data (*datNetwork, datNeworkviz*) and Affiliation data (*datAffiliation, datAffiliationAuthor*).

The structure of the database may seem redundant, for example, the author’s biographical information (*datAuthor* and *datAuthorName*) could have been merged into one file, but the separation serves a function. This splitting enables the SSHPA system to filter out overlapping author names faster because: (i) a Vietnamese author might have his or her name written differently in different publications, and (ii) the names recorded in our database are in Vietnamese spelling which has some digraphs and the addition of nine accent marks or diacritics.

As we now understand how the data are structured in the database of the SSHPA system, next we will examine how SSHPA can help improve control over the quality of data.

### Data quality assurance and control

The basic principle for building a good data verification process here is to ensure four intertwined layers of check are always carried out: (i) *inter-data-sources check:* different publicly accessible sources were used to cross-validate the accuracy of collected data; (ii) *inter-data-types check:* the different types of data collected were checked for coherence with one another; (iii) *inter-data-collectors check:* the data collectors involved in this study cross-checked the information collected by each other, especially contents that have raised doubts over accuracy; (iv) *random and periodic check*. In each step, every mistake would be classified either as a one-off or systematic type and corrected accordingly.

In the SSHPA system, based on the above principle, the process is divided into quality assurance, which refers to the techniques implemented prior to entering data, and quality control, which indicates the techniques implemented after data is entered to check for errors. Another way the quality of data could be improved is to spot strange pattern in the data through generating network visualization of authors or articles’ connections. The codes that are relevant to these processes can be found in Data Citation 1’s Codes for SSHPA.pdf.

### Quality assurance

The purpose of this step is to prevent bad data from ever being entered into the database in the first place. Several logic tests have been built into our semi-automatic system to help recognize suspicious authors or articles’ data. For the authors’ data, there are tests for:

- whether the name of an author already existed in the database
- the name of an author must not be blank
- if the author is Vietnamese, his/ her SSHPA ID must start with ‘v’; ‘f’ if foreign author
- if the author is female, her SSHPA ID must have the ‘f’ followed the initial ‘v’ or ‘f’; ‘m’ if male author; ‘?’ if sex is unknown
- the correct format of SSHPA ID must be ‘geography specifier + sex specifier + number’; for example: vm.1 is a Vietnamese male researcher numbered 1 or ff.1001 is a foreign female researcher numbered 1001.

For the articles’ data, there are tests for:

- whether the article of the same title already existed in the database
- the title for the article must not be blank
- the publisher and journal of the article must not be blank
- the year of article publication must fall in the range 2008-now
- fuzzy search article title for 90% similarity

Failure to meet these requirements and the system will notify or even block the data collector from moving to the next data points in some cases. The data, when being entered, will also be changed to match the format designated by the system. For example, the paragraph break, the quotation mark (“) ascii 147 code will be changed to (”) ascii 34 in the title of the articles.

### Quality control

This step is about applying the data validation tools to control the quality of data. The data validation tools include data filter, the search function (for relative and unique subjects), and the automatic data check functions. Here are some examples of these data validation tools.

Two authors with different SSHPA-IDs but same full names or middle names could easily be compared. And if they are suspected as being one person, the software can perform a three-step verification:

- Through name: Check the author’s name with all other authors with the same name in the system
- Through affiliations: Check the author with all other authors with the same affiliation
- Through publication: Check the author with all others with the same publication

Furthermore, the software could filter out the low-quality data such as:

- Authors with missing or invalid information: year of birth, sex, affiliation, article.
- Articles with no authors

A notable feature of our quality control is that our data team members have invited the Vietnamese researchers to cooperate by directly verifying their information in our database. Though we have yet to hear from all of them, the responses we got to date do raise the credibility of the open database.

### Automated construction of network data

**Figure 5:**
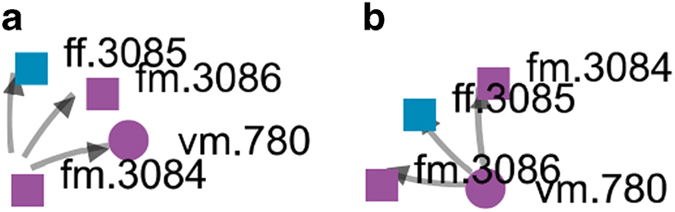
Visualizing the networks: examples. An example of both incorrect (**a**) and correct (**b**) network visualization of the data extracted from the article in 2017 by Phan *et al*.^32^. Here, each dot represents a researcher that has a connection with Phan Van Phuc, a researcher with SSHPA-ID vm.780. Purple is coded for male, blue is coded for female; the square shape represents foreign researchers while the round shape is for the Vietnamese.

**Figure 6:**
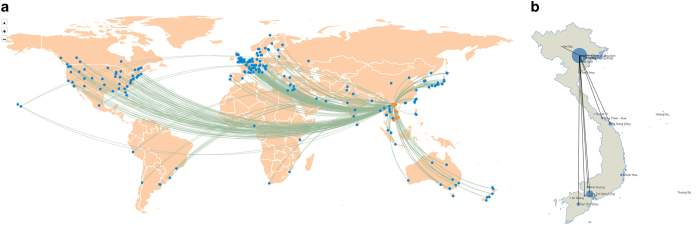
Maps of Vietnamese international and domestic scientific collaborations. (**a**) A world map of research collaborations between Hanoi, Vietnam and other places in the world. The link represents the co-authoring collaboration between Hanoi and international scholars. (**b**) A Vietnam map of the distribution of scientific publications of Vietnamese social scientists in NVSS database. The circle’s size represents the count of publications in each province; the bigger the circle the more publications. The link represents the co-authoring collaboration among scholars of each province.

**Figure 7:**
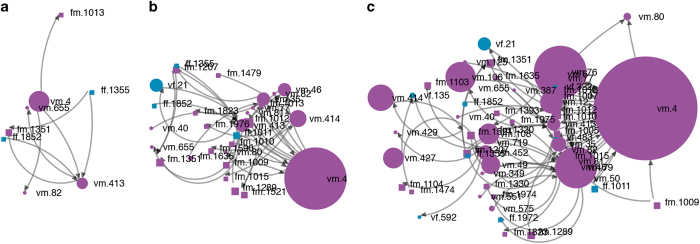
Evolution of a research group: examples of real data. The temporal evolution of a scientific group through three periods: (**a**) 2008–2010; (**b**) 2008–2014; (**c**) 2008–2018. Here, each dot represents a researcher that has a connection with vm.4. Purple is coded for male, blue is coded for female; the square shape represents foreign researchers while the round shape is for the Vietnamese. The size of the dot is the number of publications an author has within the designated period. The arrow shows the direction from key-author (first-author) to the others author in a paper.

**Figure 8:**
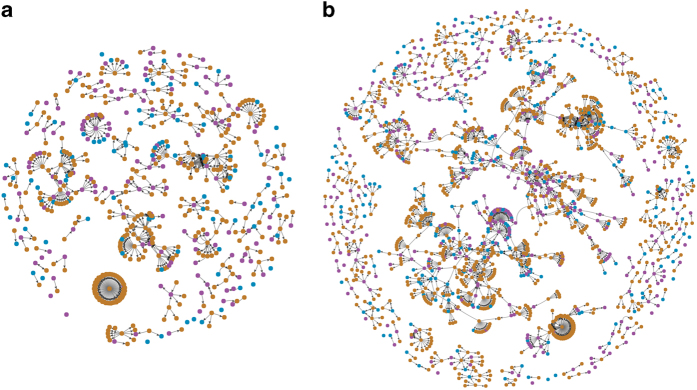
An overview of the Vietnamese scientific collaboration network. A growing network of scientific collaborations of the Vietnam’s social sciences in two periods: (**a**) 2008–2011; and, (**b**) 2008–2018. Here, each dot represents a researcher. Purple is coded for male, blue female, and orange foreign authors. The size of the dot is the number of publications an author has within the designated period. The arrow shows the direction from key-author (first-author) to the others author in a paper.

There are several kinds of network data being automatically recorded with SSHPA: co-authorship among authors (undirected network data), leading-author to non-leading author(s) connection (directed network data), co-authorship among affiliations, co-authorship among geographical locations, etc. The network data allows for different ways to representing the data visually as shown in Figs. 5, 6, 7, 8. This function enables the data collector to visualize the connections among the articles and authors in the database, thus providing him or her a new way to spot strange patterns in the data.

### Code availability

The codes that are relevant for the data quality assurance, quality control and automated construction of network data of the SSHPA system could be found in (Computer Codes, Data Citation 1).

## Data Records

The datasets are available from the *Open Science Framework* repository (Data Citation 1), under ‘NVSS 2017–18 Scientific Productivity and Collaborative Networks of Vietnamese Researchers in the Social Sciences and Humanities.’

Below is the description of the main datasets that were produced by the SSHPA system, which can be found in Data Citation 1’s Data files folder.

- ‘NVSS_Unique_articles_20180201.xlsx’ contains *N=1289* unique articles in the SSHPA database. This dataset was enabled by the use of SSHPA software’s report generation function. This dataset contains the articles’ SSHPA IDs, journal name, year of publication, authors’ names and affiliations according to each paper.
- ‘NVSS_VietnameseNodes_20180201.csv’ is a cross-section dataset containing information related to the productivity of *N=657* eligible Vietnamese authors in social sciences and humanities: id, name, year of birth, sex, number of publications in last 5 years, number of publications in leading role, number of solo publications, number of publications, contribution-adjusted productivity (‘cp’) calculated using three methods: sequence-determines-credit (cp.sdc), equal contribution for all (cp.eq) and first-last-author emphasis (cp.fl)^28,31^. The (‘cp’) results from the fact that we record the order of appearance of each author in the papers in our database; this allow us to calculate different variants of ‘cp’ with ease and flexibility. This dataset was extracted from the SSHPA database on 1^st^ February 2018.
- ‘NVSS_AllNodes_20180201.csv’ is the attribute dataset containing the data on number of publications, nationality, sex of *N=1639* authors, Vietnamese and international. It was extracted from the SSHPA database on 1^st^ February 2018.
- ‘NVSS_DirectedLinks_20180201.csv’ is a network dataset containing the data that represent the directed co-authorship connection among *N=1639* authors, Vietnamese and foreign, in the SSHPA database. It was extracted from the SSHPA database on 1^st^ February 2018.
- ‘NVSS_UnDirectedLinks_20180201.csv’ is a network dataset containing the data that represent the directed co-authorship connection among *N=1639* scholars, Vietnamese and foreign, in the SSHPA database. It was extracted from this semi-automated database on 1^st^ February 2018.
- ‘Articles and Fields 20180201.xlsx’ contains *N=1289* unique articles and their respective fields. This dataset also contains the articles’ SSHPA IDs, articles’ titles, fields, journal name, year of publication, authors’ names and their ids in the database. It was extracted from the database on 26^th^ April 2018 based on stored data until 1^st^ February 2018.
- ‘NVSS science profiles examples’ is a folder that contains the two examples of authors’ scientific profiles generated by the SSHPA system and one example of the manual system.

## Technical Validation

### Solving the problem of data duplication

Thanks to cross-validating among various sources including research publication database Scopus, our SSHPA software found that of 34,629 articles indexed by Scopus open database, 463 articles have completely similar author names, which include (seemingly) Vietnamese full names and abbreviated names. The 5,414 authors associated with these articles turn out to have different Scopus EIDs. Many of those are of different nationalities such as Korean, Chinese, Taiwanese, etc. Moreover, given that any software would assign a unique ID to a unique name, any slightest variation in a name could yield a different ID. In this case, when looking at duplicated Scopus EIDs, we found that the most duplicated articles have up to five Scopus EIDs. Indeed, if scientific output researchers in any country set out to analyse the performances of individuals or institutions in their country, the example above shows just how difficult it is to only rely on query data directly from the Scopus open database.

In our process to construct the SSHPA database, we found that the names of Vietnamese authors in the Scopus database are often not consistent, which poses a significant cost to data verification. Solving this problem requires a system that allows comparison of all name versions for any two authors. The SSHPA system has a built-in name-generating tool: once the full name of an author is entered into our database, the software will automatically generate all possible versions of names for a Vietnamese author. For example, an author with the full name “Nguyen Ngoc Anh” could generate 12 different versions of name:

[0]: “Nguyen, Ngoc Anh”

[1]: “Nguyen, N. A.”

[2]: “Nguyen, Anh Ngoc”

[3]: “Nguyen, A. N.”

[4]: “Ngoc, Nguyen Anh”

[5]: “Ngoc, N. A.”

[6]: “Anh, Nguyen Ngoc”

[7]: “Anh, N. N.”

[8]: “Ngoc, Anh Nguyen”

[9]: “Ngoc, A. N.”

[10]: “Anh, Ngoc Nguyen”

[11]: “Anh, N. N.”

Searching in the Scopus database all these versions of this name gave us 32 results. As we filtered by author name and affiliation, the search result gave two Scopus profiles of a person named ‘Nguyen Ngoc Anh’ who worked at the Development and Policies Researcher Center, Hanoi, Vietnam. In our system, this mistake is eliminated and Mr Nguyen Ngoc Anh’s profile is managed under one ID, the SSHPA-ID vm.1 (Data Citation 1). This allows our system to track any changes in the public profiles as well as update our own changes.

This is one of many similar examples where the Scopus system creates two or more Scopus EIDs for one person. This is possible because one person can be affiliated with many organizations. In the SSHPA system, we could use the author validation tool to search for authors with similar names then using other data points such as affiliations, year of birth, fields of study, articles, etc. we could check these authors are the same person or not.

### Diversifying datasets and data reports

The SSHPA software’s ability to generate many different kinds of datasets and data reports sets it apart from the time-consuming and rigid manual system (Computer Codes, Data Citation 1). Not only does this function increase the data readability for users but it also helps the system admins detect any potential anomalies, thereby, able to improve the data quality as a whole.

The SSHPA software can generate four main kinds of report, as summarized in Table 3. Examples of the reports could be viewed in Data Citation 1’s ‘Extracted and Computed Data’ folder, in which the number of articles according to fields, institutions, journals, publishers and years is shown.

As SSHPA is expected to correct for the delay in data update present in many citation indexing databases, the system will inform users of any authors or articles that were not yet found on such services. In the example of Mr Nguyen Ngoc Anh, thanks to cross-checking with other open resources such as Google Scholar, journal websites, institution websites, etc., we were able to update four of his papers that were published in 2016 and 2017, indexed in Google Scholars but were not yet found in Scopus as of 1^st^ February 2018 (Data Citation 1). Given that Vietnamese social scientists are expected to corroborate their profiles in the SSHPA database, this information will be both accurate and up-to-date.

Similarly, to improve data quality, the system’s error report will list the missing information in the database, notifying the admins of any authors, articles, or affiliations that are duplicate or lacking details. The other two kinds of report give users a full picture of the system whenever necessary, one showing the statistical distribution of authors, articles according to gender, age, fields of study, etc., and the other providing the network data per specific requirements such as citation network or co-authorship network. An example of a general statistical report produced by SSHPA can be found in Table 4. Such reports are helpful in highlighting any abnormal data point, whether that be an incorrect count of authors, papers or affiliations.

For further details, please visit Data Citation 1’s folder ‘Extracted and Computed Data’, in which details of the number of articles according to fields, institutions, journals, publishers and years are recorded.

### Improving data visualization and research applicability

Just as the creation of data reports facilitates the validation of data quality, the SSHPA software’s ability to generate descriptive statistics of networks and their visual maps also contributes to the overall quality of the database as well as its applicability in research on scientific productivity. The next part will go over the three main features and how they could help flag a mistake during data input.

### Visualization of incorrect data entry

For the most part, given the rigorous data quality assurance and quality control, the maps of networks generated by the software are rarely prone to errors. However, in case of error, the system’s ability to visualize selected scientific groups could flag the admins of any inconsistencies. For example, Fig. 5 shows a case of both correct and incorrect network visualization of the data extracted from the article in 2017 by Phan *et al*.^32^. As the author Phan Van Phuc with SSHPA ID vm.780 is the first author, the correct network visualization should have the arrows go from him to his co-authors (fm.3086, fm.3084, fm.3085) as in Fig. 5b. When we incorrectly input the author fm.3084 as the first author, the network changes as shown Fig. 5a; the arrows now go from fm.3084 toward other co-authors. In this way, the network visualization tool helps the admins recognize when a mistake in the ordering of the authors has been made.

### A map of Vietnamese social scientists’ productivity

The datasets produced by SSHPA, such as the one in Table 5, could be used for cross-section and multivariate statistical analyses, allowing us to explore how networks of Vietnamese social scientists have grown over the years^17,18^.

### The growth of scientific groups

Another feature of the SSHPA database is allowing users to study how a group of researchers who have co-authored with each other evolves over time^17^. Figure 7 is an example. First, we chose a researcher with SSHPA ID vm.4 and set his level of connection to two (meaning the network data will be limited to researchers two walks away from vm.4). Then, we picked the time periods for comparison: from 2008 to 2010 (Fig. 7a), from 2008 to 2014 (Fig. 7b), and from 2008 to 2018 (Fig. 7c).

### Network representation of all social scientists in the database

Besides enabling the visual representation of any scientific group within the database, the built-in function of network generator could also produce the visualization of all Vietnamese scientists and 973 foreign scholars (Data Citation 1) over different periods. Figure 8 is the result of this function.

Figures 7 and 8 have different ways of representing the researchers’ geography and sex. This flexibility provides the data collectors with diverse angles in examining the data patterns. In Fig. 7, colour represents sex while shape represents geography (Vietnamese or overseas); thus, both Vietnamese and foreign authors are represented equally. Figure 8 highlights the growth of research networks between Vietnamese authors and overseas, with three colours denoting the sex of Vietnamese scholars and the status of scholars who are foreign. In this way, network visualization enables a better reading of the data, thereby, improving the quality of the open database.

## Usage Notes

As the SSHPA system relies only on open and free recourses to collect and verify data related to scientific output, it opens the possibility of replicating the same system in other places throughout the world (Computer Codes, Data Citation 1). It is also applicable for collecting and verifying data in other fields such as biomedicine, math, biology, etc. This work, therefore, aims to reduce the cost of doing science^10,33,34^.

The datasets of this study are first and foremost suitable for analyses of the relationship between individuals’ scientific output and demographic (such as sex, age, region) as well as their collaboration characteristics. Moreover, with the development of the SSHPA software, one can also get access to citation and co-authorship network data, which would allow for more in-depth analyses of scientific influence among the researchers in the datasets.

We are convinced that the practice of open data is one of many ways to help cure the crisis of reproducibility in social sciences^10,34,35^, and improve policy-making processes when it comes to the question of funding efficiency^36^. We wish for scientists all over the world working on the issue of scientific productivity to replicate the design of the SSHPA system to verify the usefulness of the system in cleaning and eliminating data errors.

## Additional information

**How to cite this article**: Vuong, Q. H. *et al*. An open database of productivity in Vietnam's social sciences and humanities for public use. *Sci. Data* 5:180188 doi: 10.1038/sdata.2018.188 (2018).

**Publisher’s note**: Springer Nature remains neutral with regard to jurisdictional claims in published maps and institutional affiliations.

## Supplementary Material



## Figures and Tables

**Table 1:** Input names and explanation.

	**Input name**	**Variable name**	**Explanation**
1	Age	age	The age of the survey subject
2	Sex	sex	The sex of the survey subject
3	Affiliation	affil	During the manual phase, we chose the affiliations where the researchers are full-time employed. During the semi-automatic phase, due to the data structure, we assigned the affiliations according to each paper.
4	Region	reg	A region is specified by the affiliations of the researchers. There are four categories: North, South, Centre and Overseas.
5	Fields of study	field	During the manual phase, we chose the field registered as their major in PhD or Master degree. During the semi-automatic phase, due to the data structure, we assigned the fields of study according to each paper.
6	Total items	ttitems	The total number of publications in Scopus. Unit: item(s).
7	Solo author	au.solo	The number of solo publications. Unit: item(s).
8	Key author	au.key	The number of publications the survey subject is in leading role. A person is considered to have a leading role in an article when he or she served as the solo author, the first author or the corresponding author. Unit: item(s).
9	Co-author	au.co	The number of publications where the survey subject is neither leading author nor solo author. Thus, *au*.*co*=*ttlitems−au.key*. Unit: item(s).
10	Contribution-adjusted productivity	cp: cp.sdc, cp.eq, cp.fl	The measure of relative scientific output. When we calculate this measure *manually*, the absolute productivity is converted using the method of sequence-determines-credit (cp.sdc)^29–31^. Then, using the *semi-automatic system*, we added two more measures: equal contribution for all (cp.eq) and first-last-author emphasis (cp.fl)^29,31^.
11	Vietnamese author	au.vn	The number of Vietnamese researchers a survey subject has co-authored with. Each Vietnamese researcher is counted only once. Unit: people.
12	Foreign author	au.fr	The number of foreign researchers a survey subject has co-authored with. Each foreign researcher is counted only once. Unit: people.
13	Unique author	au.uniq	The sum of au.vn and au.fr. Unit: people

**Table 2:** SSHPA’s descriptive statistics on the productivity of Vietnamese researchers in SS&H from 2008 to 2018.

**Variables**	**Min**	**Max**	**Mean**	**Median**	**SD**	**5 to 95 percentiles**
last5year	0	34	2.164	1.00	3.564	(1.891, 2.437)
age	20	76	43.38	41.00	9.743	(42.588, 44.172)
au.solo	0	61	0.510	0.00	2.771	(0.298, 0.722)
au.vn	0	40	1.904	1.00	3.135	(1.664, 2.144)
au.fr	0	50	1.903	1.00	3.536	(1.632, 2.173)
ttlitems	1	67	3.212	1.00	5.276	(2.807, 3.616)
cp.sdc	0.1	65.2	2.013	1.00	4.090	(1.700, 2.327)
cp.eq	0.1	63.4	1.322	0.50	3.329	(1.066, 1.577)
cp.fl	0.1	67	2.297	1.00	4.420	(1.959, 2.636)
The statistics in Table 2 can be used to derive some useful reports on the number of authors, foreign co-authors, published papers, research institutions, as well as calculations of distributions of publications over authors (groups of authors), institutions, publications solely by Vietnamese authors, most productive field of research, etc.						

**Table 3:** Varieties of reports which could be produced with SSHPA.

**Report types**	**Subjects**	**Details**
Comparative Report	SSHPA and other scientific citation indexing services	Reporting authors or articles that could not be found on other open research publication databases Reporting differences in update time, authors number, article numbers, etc.
Error report	Authors	Lacking information (year of birth, gender, age, affiliations, etc.) Duplicate records (full or partial duplication)
	Articles	Lacking information (publisher, journal, year of publication, etc.) Duplicate records (full or partial)
	Affiliations	Lacking information (province, city, country, etc.) Duplicate records (full or partial)
Statistics report	Authors	Statistical distribution according to gender, age, geographical locations, etc.
	Articles	Statistical distribution according to fields of research, institutions, geographical locations, etc.
	Affiliations	Distribution of articles and authors according to affiliations
Network Statistics Report	Co-authorship	Network data of all Vietnamese social sciences scholars in SSHPA database (with or without direction, weighted or non-weighted) Network data of scholars in one or many institutions (with or without direction, weight of non-weighted)
	Citation network	Network data of citation among people (with direction, weighted or non-weighted) Network data of citation among articles (with direction, weighted or non-weighted)

**Table 4:** Statistical reports produced by SSHPA.

**Description**	**Quantity**	**Unit**
Total number of eligible Vietnamese SS&H authors	657	author
Total number of eligible foreign co-authors	973	author
Total number of papers published	1,289	article
Total number of affiliations (Vietnamese and foreign)	743	affiliation
% of unique papers belong to top 10 researchers	22.73	%
% of unique papers belong to top 5 affiliations	34.13	%
% of unique papers published by Vietnamese only	41.19	%
% of unique papers published with foreign authors	58.81	%
% of single-authored unique papers	26.14	%
Vietnamese researcher with most publications	67	article
Vietnamese researcher with most solo publications	61	article
Vietnamese institution with most publications	147	article
Journal with most publications by Vietnamese	17	article
Publisher with most publications by Vietnamese	242	article
Researcher with the most co-authors	36	author
Field with most publications	366	article

**Table 5:** Descriptive statistics of networks generated by SSHPA.

**Figures’ network**	**Figure 5a: World map 2008**–**2018**	**Figure 7a: Network of all 2008**–**2011**	**Figure 7b: Network of all 2008**–**2018**
**Directed or not**	**Undirected**	**Directed**	**Directed**
**Nodes**	287	574	1,624
**Edges**	4610	587	1,914
**mean degree**	32.125	2.045	2.357
**transitivity**	0.616	0.077	0.121
**reciprocity**	NA	0.051	0.103
Nodes in the largest component	285	58	782
Together with the numerical understanding of networks, SSHPA can also provide a visual representation of the collaboration patterns geographically, among Vietnamese provinces or between Vietnam and the world (See Fig. 6).			
